# Supplementary material for: MetaQTL: a package of new computational methods for the meta-analysis of QTL mapping experiments
Source: BMC Bioinformatics. 2007 Feb 8;8:49. doi: 10.1186/1471-2105-8-49 (PMC1808479; doi:10.1186/1471-2105-8-49)
Supplement: Additional File 7 — MetaQTL Package : jar file and tutorial. This Zip archive contains both the MetaQTL JAR file and the files of the tutorial. [file 1471-2105-8-49-S7.zip › org.inra.metaqtl/doc/org/metaqtl/main/MQTLView.html]

MQTLView


|  |  |  |
| --- | --- | --- |
| **Overview** \| **Package** \| **Class** \| **Use** \| **Tree** \| **Deprecated** \| **Index** \| **Help** | |  |
| **PREV CLASS**   **NEXT CLASS** | **FRAMES**    **NO FRAMES**     **All Classes** |  |
| SUMMARY: NESTED \| FIELD \| CONSTR \| METHOD | DETAIL: FIELD \| CONSTR \| METHOD |  |


---


## org.metaqtl.main Class MQTLView

```
java.lang.Object
  org.metaqtl.main.MetaMain
      org.metaqtl.main.MQTLView
```

---

``` public class MQTLView extends MetaMain ```

---

**Field Summary**

**Fields inherited from class org.metaqtl.main.MetaMain**

`help, parser, verbose`


**Constructor Summary**

`MQTLView()`


| **Method Summary** | |
| --- | --- |
| `static void` | `main(java.lang.String[] args)` |
| `void` | `printHelp()` |
| `void` | `printUsage()` |

**Methods inherited from class org.metaqtl.main.MetaMain**

`generalHelp, generalUsage, getChromByNames, getChromGraph, getCluster, getClusterByNames, getDico, getDubious, getMap, getMap, getMaps, getMrkToRm, getOntology, getQtlPartition, getQtlPartition, getResult, initCmdLineParser, parseChromList, parseCmdLine, printImgFormat, printLicense, removeLocus, setParameter, synchChromGraphs, writeImg, writeMap, writeMap, writePar`

**Methods inherited from class java.lang.Object**

`clone, equals, finalize, getClass, hashCode, notify, notifyAll, toString, wait, wait, wait`

**Constructor Detail**

### MQTLView

```
public MQTLView()
```


**Method Detail**

### printUsage

```
public void printUsage()
```

:   **Specified by:**: `printUsage` in class `MetaMain`

---


### printHelp

```
public void printHelp()
```

:   **Specified by:**: `printHelp` in class `MetaMain`

---


### main

```
public static void main(java.lang.String[] args)
```


---


|  |  |  |
| --- | --- | --- |
| **Overview** \| **Package** \| **Class** \| **Use** \| **Tree** \| **Deprecated** \| **Index** \| **Help** | |  |
| **PREV CLASS**   **NEXT CLASS** | **FRAMES**    **NO FRAMES**     **All Classes** |  |
| SUMMARY: NESTED \| FIELD \| CONSTR \| METHOD | DETAIL: FIELD \| CONSTR \| METHOD |  |


---
